# Industrial Product Surface Anomaly Detection with Realistic Synthetic Anomalies Based on Defect Map Prediction

**DOI:** 10.3390/s24010264

**Published:** 2024-01-02

**Authors:** Tao Peng, Yu Zheng, Lin Zhao, Enrang Zheng

**Affiliations:** 1 School of Electrical and Control Engineering, Shaanxi University of Science and Technology, Xi’an 710026, China; 210611013@sust.edu.cn (T.P.); 210612064@sust.edu.cn (L.Z.); 2 School of Cyber Engineering, Xidian University, Xi’an 710126, China; yuzheng.xidian@gmail.com

**Keywords:** defect detection, image reconstruction, synthetic anomalies, defect separation

## Abstract

The occurrence of anomalies on the surface of industrial products can lead to issues such as decreased product quality, reduced production efficiency, and safety hazards. Early detection and resolution of these problems are crucial for ensuring the quality and efficiency of production. The key challenge in applying deep learning to surface defect detection of industrial products is the scarcity of defect samples, which will make supervised learning methods unsuitable for surface defect detection problems. Therefore, it is a reasonable solution to use anomaly detection methods to deal with surface defect detection. Among image-based anomaly detection, reconstruction-based methods are the most commonly used. However, reconstruction-based approaches lack the involvement of defect samples in the training process, posing the risk of a perfect reconstruction of defects by the reconstruction network. In this paper, we propose a reconstruction-based defect detection algorithm that addresses these challenges by utilizing more realistic synthetic anomalies for training. Our model focuses on creating authentic synthetic defects and introduces an auto-encoder image reconstruction network with deep feature consistency constraints, as well as a defect separation network with a large receptive field. We conducted experiments on the challenging MVTec anomaly detection dataset and our trained model achieved an AUROC score of 99.70% and an average precision (AP) score of 99.87%. Our method surpasses recently proposed defect detection algorithms, thereby enhancing the accuracy of surface defect detection in industrial products.

## 1. Introduction

Defects on the surface of industrial products refer to incomplete, irregular, or non-compliant areas or traces that occur during manufacturing, processing, or usage. These defects can be caused by physical, chemical, mechanical, or other factors and they can affect the appearance, quality, and performance of the products. The presence of defective products has a significant impact on both businesses and users. In mature industrial production processes, defective products exhibit three main characteristics. Firstly, the number of defective products is extremely low compared to normal products. Secondly, the defects exhibit various forms and diverse types. Thirdly, the defect areas are relatively small and the defect images are similar in distribution to the normal images. Therefore, identifying the differences between normal and defective samples is a highly challenging task.

Traditional detection methods primarily rely on increased allocation of human resources, where product quality inspectors visually discern the quality of products. This approach proves to be inefficient and incurs high costs. In addition, machine vision-based defect detection methods have also been widely explored, including techniques such as edge detection, threshold segmentation, and texture analysis. However, these techniques exhibit significant limitations when applied. For example, noise and variations in illumination can directly result in inaccurate edge detection, unstable threshold segmentation, and interference with the texture analysis results. Moreover, these methods typically rely on designed feature extraction, lacking good adaptability to different types of defects or image scenes, requiring adjustments and optimizations specific to the problem at hand, which further involves the challenge of parameter selection. In recent years, there has been rapid progress in deep learning methods aimed at emulating human habits and capabilities, with the objective of substituting humans in performing complex and high-risk tasks. With the swift advancement of computer technology and the enhancement of computational capabilities, the performance of deep learning-based anomaly detection techniques has been continuously improving. These techniques have found extensive applications in various domains, including agricultural production [1,2], industrial manufacturing [3,4], aerospace [5,6], and computer network security [7,8].

Supervised anomaly detection based on image data is one of the commonly employed methods in the field of deep learning. By being able to learn the distinctive features of positive and negative samples, it typically achieves the desired task objectives. However, the stable performance of supervised learning methods relies on a massive dataset with a balanced distribution of positive and negative samples. The major challenge in surface defect detection tasks lies in the extremely limited quantity of defect samples, which can result in overfitting of the model during fully supervised learning and subsequently affects the detection accuracy. In comparison, reconstruction-based semi-supervised anomaly detection methods, which do not require labeled defect samples, have gained popularity as an alternative approach. Among them, the two most classical categories are based on Generative Adversarial Networks (GANs) and Autoencoders (AEs), two fundamental techniques in the field of semi-supervised learning for image reconstruction. These methods extensively train on a large number of normal samples, aiming to learn the close relationship between the high-dimensional and low-dimensional distributions of images. This enables the network to learn how to reconstruct output images that closely resemble the input images. During testing, defect images are fed into the pre-trained network model, and due to significant differences from the reconstructed images, they are effectively identified and filtered out. Therefore, reconstruction-based anomaly detection methods have become an effective means to accomplish surface defect detection tasks in industrial products. When the network is trained to be too robust, it tends to perfectly reconstruct defect images as well, thus evading detection.

However, this type of image reconstruction technique is trained only using normal samples, and real defect images have never been involved in the entire process. This makes the inference of the entire network somewhat biased. The reality is that the scarcity of real defect images prevents their inclusion in the training process, and artificially synthesized defects generally differ significantly from real defects. As a result, the trained network exhibits poor generalization ability and fails to detect real defective products. Additionally, the authenticity of the reconstructed images serves as a criterion for assessing the performance of the reconstruction network. While autoencoders primarily focus on the reconstruction effect on high-dimensional images without considering low-dimensional features, Ganomaly takes into account the reconstruction consistency of low-dimensional latent vectors. However, training Ganomaly [9] is often challenging and struggles to converge to the global optimum.

In response to the aforementioned issues, this study was inspired by the DRAEM [10] concept to create more realistic and plausible synthetic anomaly images. This approach addresses the problem of defect images not being involved in the training process. An image reconstruction network was designed with deep feature consistency, and the network’s ability to separate defects was enhanced by utilizing the larger effective receptive field provided by the use of oversized convolutional kernels. This resulted in the generation of defect region prediction maps. By calculating the loss function using the predicted maps and the real defect regions, the possibility of the network model directly reconstructing defect images was eliminated, thus achieving more accurate surface defect detection in industrial products. The main contributions of this study are as follows:

- A methodology for creating more realistic synthetic defect images is designed.
- An image reconstruction network with depth feature consistency is constructed.
- A defect prediction network with a widely effective receptive field is being constructed.

## 2. Related Work

### 2.1. The Study of Anomaly Synthesis

**Figure 1:**
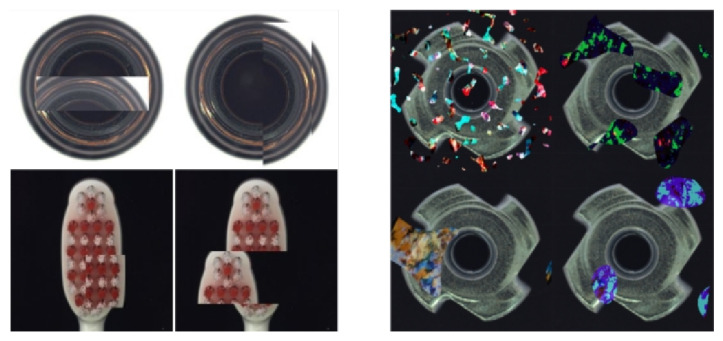
The left-hand side of the figure presents an example of defect synthesis using the CutPaste method, while the right-hand side shows an example of defect synthesis using the DRAEM approach.

Obtaining a large amount of defect data is a very challenging issue in defect detection tasks. Synthetic anomaly is a reverse solution approach that addresses this challenge by artificially creating more anomalous situations and expanding the defect dataset. The CutPaste method proposed by Chung-Liang Li et al. [11] has been validated on the MVTec [12] dataset. This method involves cutting out patch blocks from images and pasting them randomly onto the image to augment the dataset. This data augmentation strategy is simple and effective, enabling the model to detect local irregularities of the target. However, this random masking method for creating anomalies does not match actual situations. For instance, in the bottle dataset, the edge of the bottle bottom may appear in the middle of the bottle image, and in the toothbrush dataset, the top of the toothbrush head may appear in the middle of the toothbrush head (as shown on the left in Figure 1). The FIP method proposed by Jeremy Tan et al. [13] extracts the same patch area from two independent samples, uses interpolation between the two patches to obtain a fused patch, and then replaces it at the original patch position. The model trained with this method has stronger generalization ability and can detect subtle irregularities, performing well on the MOOD Challenge [14] dataset of medical images. NSA [15] uses Poisson image editing to make the synthesized defects more natural and closer to real anomalies. DRAEM first uses Berlin noise to crop DTD [16] texture dataset images and then paste them onto the images to be trained. The design of the discriminative network is specifically for learning the ability to separate these synthesized anomalies. However, the Berlin noise is superimposed on the entire image, beyond the scope of the foreground target (as shown on the right in Figure 1) and differs significantly from real anomalies, resulting in inaccurate defect positioning.

### 2.2. The Study of Defect Detection

Image reconstruction has recently been widely used for anomaly detection. Although it was not originally designed for anomaly detection, it can be forced to capture key underlying patterns through learning the representation of data instances. AnoGAN [17] was the first method to apply GAN [18] to anomaly detection. During the inference stage, AnoGAN requires a huge amount of computational resources to iteratively search for the latent vector z corresponding to an input instance X. Ganomaly, proposed later, improved upon AnoGAN by incorporating an encoder, which learns the ability to transform image instances into latent space vectors during the training process and detects anomalies by calculating the distance between the input image and the reconstructed image. Convolutional Autoencoders are also widely used for data compression and dimensionality reduction. Comprising of an encoder and a decoder, the network model must retain the essential information of data instances to minimize the reconstruction error. DRAEM adopts a dual autoencoder architecture and uses a re-embedding technique to directly learn the anomaly distance function, achieving good performance in anomaly detection.

The flow-based method was initially used for network traffic analysis and security monitoring. Recently, with the development of computer technology, the algorithm performance has been significantly improved. Cflow [19], Csflow [20], and Fastflow [21] determine anomalies by analyzing the characteristic patterns in data flows and using unsupervised methods to learn anomaly patterns from the data. They have strong adaptability to the data, but Cflow can only detect abnormal traffic significantly different from normal data, as Csflow has weak processing ability for high-dimensional data, which can result in false positives or negatives, and Fastflow has limited effectiveness in industrial product defect detection due to the need for a large amount of data for training and weak processing ability for high-dimensional data.

Using pre-trained models can greatly reduce training time and have good feature extraction capabilities. STFPM [22] and RDFOCE [23] are based on the teacher–student network architecture and belong to a class of knowledge distillation methods that cooperate with pre-trained models. They can be trained end-to-end, but RDFOCE requires a high amount of training data, as insufficient training data can lead to performance degradation. STFPM may perform poorly when dealing with large-sized images due to the large amount of data needed.

Performing data feature extraction followed by processing the feature set is also a good approach for anomaly detection. PatchCore [4] divides images into patches, extracts features via convolutional networks, learns the similarity of nodes in the PatchCore graph, and detects anomalies using clustering. PaDim [24] shares a similar approach with PatchCore, but uses an anomaly detection model to detect anomalies. DFM [25] also extracts features to establish the probability distribution of normal samples in the feature space and detects anomalies by calculating the likelihood of a new sample belonging to normal samples. The commonality among these three methods is that they rely too much on the accuracy of the feature extraction network. If there are few available normal samples for learning, it may lead to problems such as feature learning bias. In addition, other methods include CFA [26], which uses feature adaptation and coupled hypersphere methods for anomaly detection, but consumes significant computational resources.

## 3. Method

**Figure 2:**
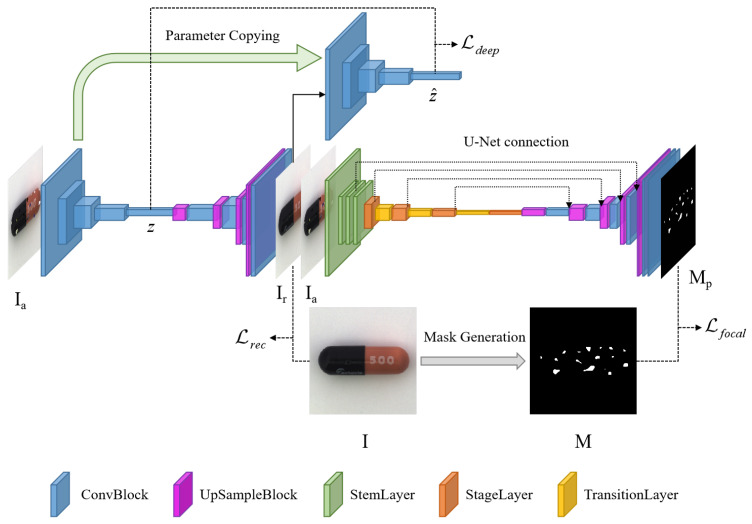
The model consists of a reconstruction network on the left and a defect prediction network on the right. The reconstruction network comprises an autoencoder and a deep feature extractor, while the defect prediction network employs an ultra-large kernel convolutional encoder and connects the encoding and decoding components via a U-Net network.

The defect detection algorithm model proposed in this study, which is based on the prediction of defect maps through the learning of abnormal distance function, is composed of an image reconstruction network and an anomaly separation network (as shown in Figure 2).

The image reconstruction network is trained to ensure that the reconstructed image and the original normal image have highly similar high-level semantic information and low-level semantic information, resulting in high visual similarity between the two. The anomaly separation network takes the reconstructed image and the synthesized abnormal image as inputs and aims to learn the distance function between the abnormal image and the real image, thereby generating accurate abnormal segmentation images and completing the defect detection task. The mechanism for synthesizing anomalies adopts a simple cut-and-patch method to mimic real anomalies and add a large number of realistic defect samples, thus compensating for the sample imbalance problem caused by the lack of defect images in the training data of the image reconstruction method.

### 3.1. Abnormal Synthesis Process

**Figure 3:**
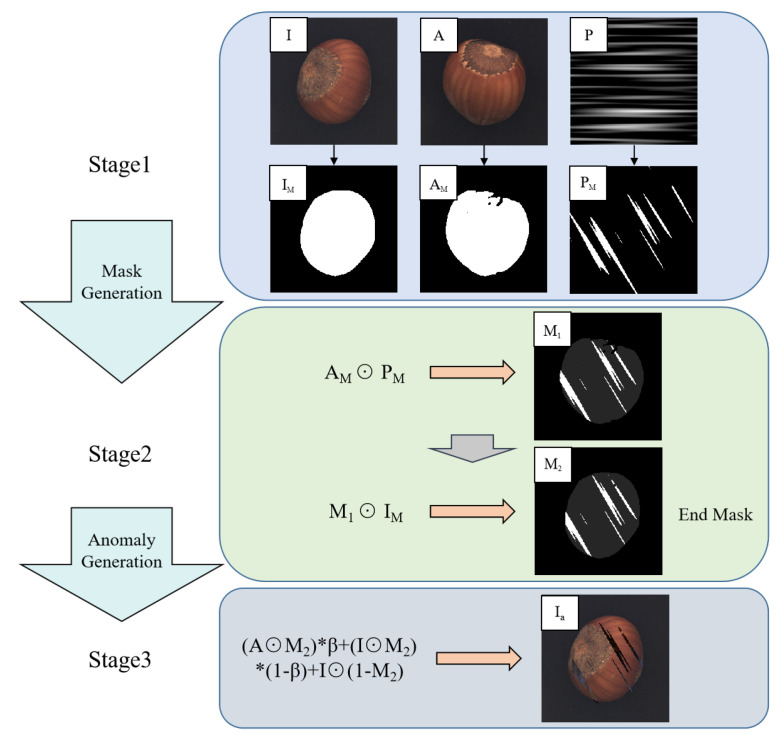
The three stages of anomaly image synthesis.

Defects can be commonly understood as the situation where the contextual information of a certain region on the foreground target is significantly different from that of the surrounding areas and is unrelated to the target background. Unlike DRAEM, we emphasize the authenticity of synthesizing anomalies. Based on this principle, the process of generating synthetic abnormal images can be divided into three stages (as shown in Figure 3).

In the first stage, an input image *I* is selected and a sample *A* is randomly extracted from the normal images in the same dataset to serve as the anomaly source. The foreground object corresponding to the region is obtained by using edge detection with dilated padding or by directly setting a grayscale threshold, resulting in the corresponding mask images $I_{M}$ and $A_{M}$. We use a Perlin noise generator to generate random noise texture image *P*, which is then compared with a preset threshold to produce a binary mask image $P_{M}$.

In the second stage, since *P* is randomly generated, the unobstructed areas of $P_{M}$ (the white area of $P_{M}$ in Figure 3) may appear within the specified range (the size of the image), but we want the synthetic anomaly to appear on the foreground object. Therefore, the anomaly source mask image $A_{M}$ is first multiplied pixel-wise with the Perlin noise mask image $P_{M}$ to obtain the mask image $M_{1}$, and the defect region is constrained within the valid range. Then, the input image mask image $I_{M}$ is multiplied pixel-wise with $M_{1}$ to obtain the final mask image $M_{2}$ (the same as *M* in Figure 2). Therefore, the final mask image $M_{2}$ is defined as:

(1) $$M_{2}=A_{M}⨀P_{M}⨀I_{M}$$

**Figure 4:**
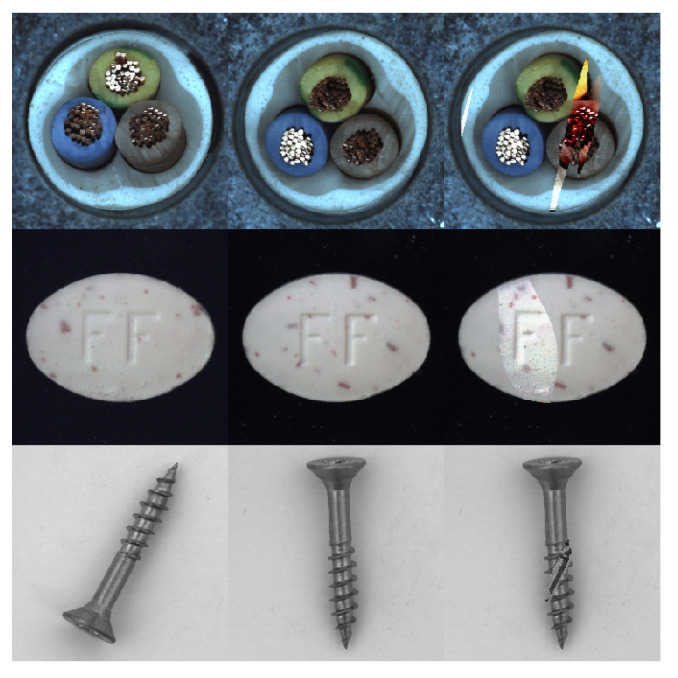
From left to right, the three columns are the anomaly source image *A*, the input image *I*, and the synthesized anomaly image $I_{a}$.

In the third stage, $M_{2}$ is used to extract a portion of the region from sample *A*, and similarly, $M_{2}$ is used to extract the corresponding region from input image *I*, which is then blended using random interpolation to obtain the final defect image. It is then combined with the other regions $\left(1-M_{2}\right)$ of the input image *I* to obtain the final synthesized anomaly image. Therefore, the anomaly image $I_{a}$ is defined as:

(2) $$I_{a}=\left(A⨀M_{2}\right)\beta +\left(I⨀M_{2}\right)\left(1-\beta \right)+I⨀\left(1-M_{2}\right)$$

where ⨀ is pixel-wise multiplication, while β is a random interpolation coefficient with β∈[0,0.8). The defect region created using the random interpolation blending method includes both the partial information of the original image *I* and the information from the anomaly source image *A*, which makes the synthesized anomaly diverse and realistic. Figure 4 presents a set of examples of synthesized anomaly images.

Therefore, our synthetic anomaly method ensures that the anomaly cases appear only on the foreground object, independent of the background, and the anomalies produced are more realistic.

### 3.2. Image Reconstruction Network

The reconstruction module consists of an autoencoder and a deep feature vector extractor, which aim to extract key information from synthesized defective images and reconstruct the original image (as shown on the left in Figure 2) using the reconstruction network. The network structure of the deep feature vector extractor is identical to the encoder part of the autoencoder but does not participate in network parameter updates. Instead, before each training session, all the parameters of the encoder are copied to the corresponding locations of the feature extractor. The intuition behind this design is that the entire reconstruction network, constrained by both the reconstruction loss function and deep feature loss function, can learn to reconstruct normal images or synthesized anomaly images into normal images via continuous training. In other words, the encoder part of the autoencoder can extract key information for perfect reconstruction from different input images, and its ability to extract key features continues to improve. Therefore, it is reasonable to use the feature extractor with the same parameter settings to extract deep features for the reconstructed image.

The $L_{2}$ loss function is commonly employed to compute the sum of squared pixel differences between generated and real images. However, it is heavily influenced by noise and outliers and exhibits poor recovery performance for edge details. The $L_{2}$ loss is defined as follows:

(3) $$L_{2}\left(I,I_{r}\right)=\frac{1}{HW}\sum_{i=1}^{H}\sum_{j=1}^{W}{∥I_{a}\left(i,j\right)-I\left(i,j\right)∥}^{2}$$

The SSIM [27] loss function can be used to measure the structural similarity between the generated image and the original image and can compensate for the shortcomings of the $L_{2}$ loss function. The SSIM loss is defined as follows:

(4) $$L_{SSIM}\left(I,I_{r}\right)=\frac{1}{HW}\sum_{i=1}^{H}\sum_{j=1}^{W}1-SSIM{\left(I,I_{r}\right)}_{\left(i,j\right)}$$

The variables *H* and *W* in Equations (3) and (4) represent the height and width of the input image *I*, respectively, which denotes the reconstructed image generated by the network, and SSIM is the similarity function used to measure the similarity between *I* and $I_{r}$.

The two loss functions are combined proportionally to form the visual image reconstruction loss function $L_{vision}$, which is used to measure the loss of image reconstruction in terms of visual perception.

(5) $$L_{vision}\left(I,I_{r}\right)=\lambda_{1}L_{SSIM}\left(I,I_{r}\right)+L_{2}\left(I,I_{r}\right)$$

where $\lambda_{1}$ is a hyperparameter used to balance the two loss functions.

In addition, the loss function $L_{1}$ is calculated based on the deep feature vectors of the extracted input image *z* and the reconstructed image $\hat{z}$, in order to ensure that the generated image is close to the original one in terms of high-level semantic information. This part of the loss is defined as $L_{deep}$.

Therefore, the loss function of the image reconstruction network is formulated as follows:

(6) $$L_{rec}\left(I,I_{r}\right)=\lambda_{2}L_{vision}\left(I,I_{r}\right)+\lambda_{3}L_{deep}\left(z,\hat{z}\right)$$

where $\lambda_{2}$ and $\lambda_{3}$ are hyperparameters used to balance the visual loss and deep feature loss, respectively, in the loss function of the image reconstruction network.

### 3.3. The Large Convolutional Kernel Defect Prediction Network

The RepLKNet network proposed by Xiaohan Ding et al. [28] uses a large 31 × 31 convolutional kernel for computation, which has a larger effective receptive field compared to the approach of using multiple small convolutional kernels to form an equivalent large one, demonstrating good performance on ImageNet [29] classification, COCO [30] detection, and ADE20K [31] segmentation tasks. The defect prediction network adopts an autoencoder architecture and employs U-Net [32] network connections (as shown on the right in Figure 2). The reconstructed image $I_{r}$ and the synthesized abnormal image $I_{a}$ are concatenated at the channel level and inputted into the network. The network learns an appropriate distance metric between the reconstructed image $I_{r}$ and the input abnormal image $I_{a}$, predicting the probability of defects occurring at the pixel level. The design concept of using large convolutional kernels is employed in the encoder part of the network, where the concatenated image $I_{r}+I_{a}$ is inputted with a size of 256 × 256 and six channels. After being processed via four stem layers, the output is a feature map with 128 channels and a size of 64 × 64. The feature map then enters the stage block, which includes four stages that use large convolutional kernels of sizes [31, 29, 27, 13] to extract information. To address the optimization problems, the small kernel reparameterization is introduced. The synthesized defects are generated using Gaussian noise, and the distribution of the abnormal areas is random, resulting in an imbalance of the defect and normal areas. Focal Loss [33] has shown good performance in dealing with sample imbalance and difficult classification problems. Therefore, it is selected as the loss function $L_{seg}$ for the defect prediction network:

(7) $$L_{seg}=L_{focal}\left(p_{t}\right)=-\alpha_{t}{\left(1-p_{t}\right)}^{\gamma }\log\left(p_{t}\right)$$

where $p_{t}$ is defined as:

(8) $$p_{t}=\left\{\begin{array}{cc} p, & if\;y=1\\ 1-p, & otherwise \end{array}\right.$$

In our model, *p* represents the probability that each pixel position in the predicted abnormal image outputted by the defect prediction network is an abnormal area.

Taking into account the two parts mentioned above, the overall loss function $L_{total}$ of the network is formulated as follows:

(9) $$L_{total}\left(I,I_{r},M_{2},M_{p}\right)=L_{focal}\left(p_{t}\right)=L_{rec}\left(I,I_{r}\right)+L_{seg}\left(M_{2},M_{p}\right)$$

where $M_{2}$ is the final mask image, representing the ground truth, and $M_{p}$ is the defect prediction image.

### 3.4. Abnormality Score

The defect prediction image $M_{p}$ can serve as a criterion for judging whether there are abnormalities. After being smoothed via mean filtering to aggregate local abnormal information, the final image-level abnormality score is obtained by utilizing maximum pooling:

(10) $$\eta =max(M_{p}*f_{s_{f}\times s_{f}})$$

where ∗ represents the convolutional operator, $f_{s_{f}\times s_{f}}$ is a mean filter with a size of $s_{f}\times s_{f}$, max is the maximum pooling operation, and the abnormality score η corresponds to the maximum value in the feature map after maximum pooling.

## 4. Experiments

The performance of this method was evaluated and compared with other advanced methods in the field of defect detection. Furthermore, the effectiveness of each component module of the proposed method was validated via ablation experiments.

### 4.1. Experimental Setup

We evaluated our method on the MVTec anomaly detection dataset, which is currently a challenging benchmark test set used to evaluate and compare different defect detection algorithms. MVTec AD contains approximately 5000 real industrial defect images from 15 different categories in 13 industrial sectors, including approximately 2500 defective images. The dataset also provides pixel-level mask annotations to indicate the location and shape of the defects in the images. In anomaly detection, image-level AUROC is commonly used to evaluate the algorithm’s ability to detect anomalies. To evaluate the performance of our proposed method, we used image-level AUROC as an evaluation metric in anomaly detection. Additionally, we also used average precision (AP) as a benchmark for evaluating the model’s ability to locate defects.

In the experiment, we trained the network on the MVTec AD dataset for 700 epochs, with a learning rate set to 10${}^{-4}$. We performed fine-tuning by multiplying the learning rate by 0.1 at 400 and 600 epochs to achieve global optimization. Throughout the training process, we saved the best-performing model. The hyperparameters in the loss function were set to $\lambda_{1}=1$, $\lambda_{2}=0.8$, and $\lambda_{3}=0.2$, respectively.

During training, we also used data augmentation via image rotation to compensate for the limited number of training samples. We still used MVTec AD as a source of anomaly images for defect manufacturing to create more realistic defect images and improve the model’s robustness. The experiment was conducted on a computer equipped with an NVIDIA RTX 3090 GPU.

### 4.2. Anomaly Detection

**Figure 5:**
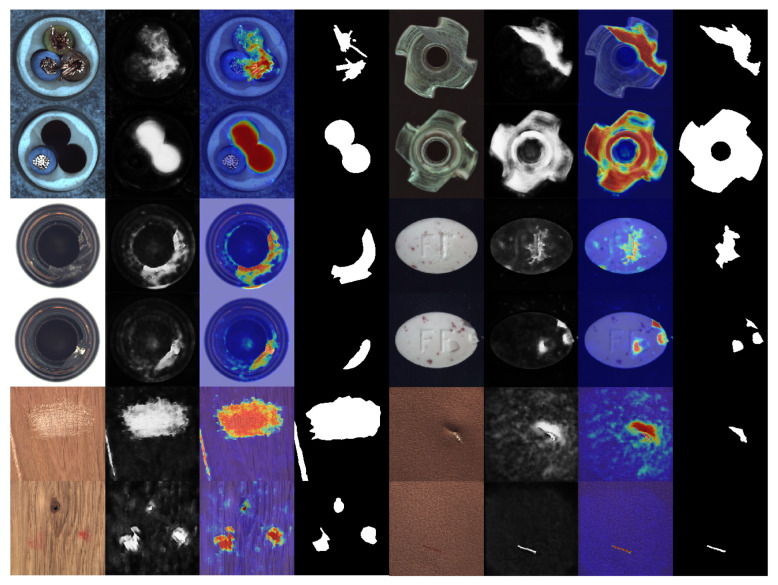
Results of defect prediction for several categories. For each category, the four images from left to right are the original image, the defect prediction image, the heat map, and the ground truth.

**Figure 6:**
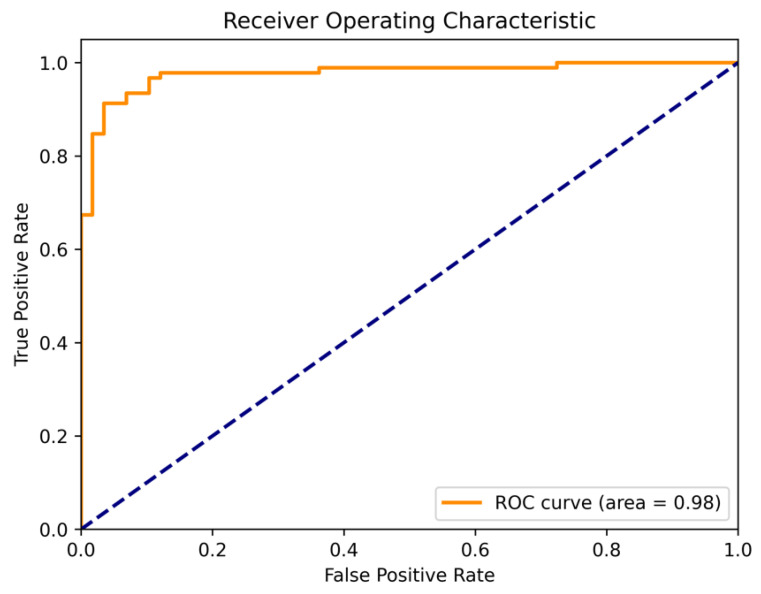
The ROC curve for the cable dataset is shown in the upper and lower halves of the figure, respectively.

**Figure 7:**
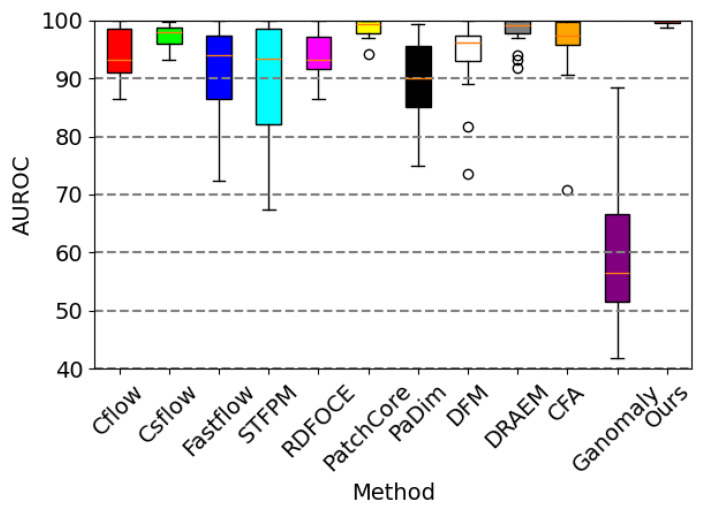
Visualizations of the box plots for Table 1 and Table 2 show the distribution of results for each method.

**Figure 8:**
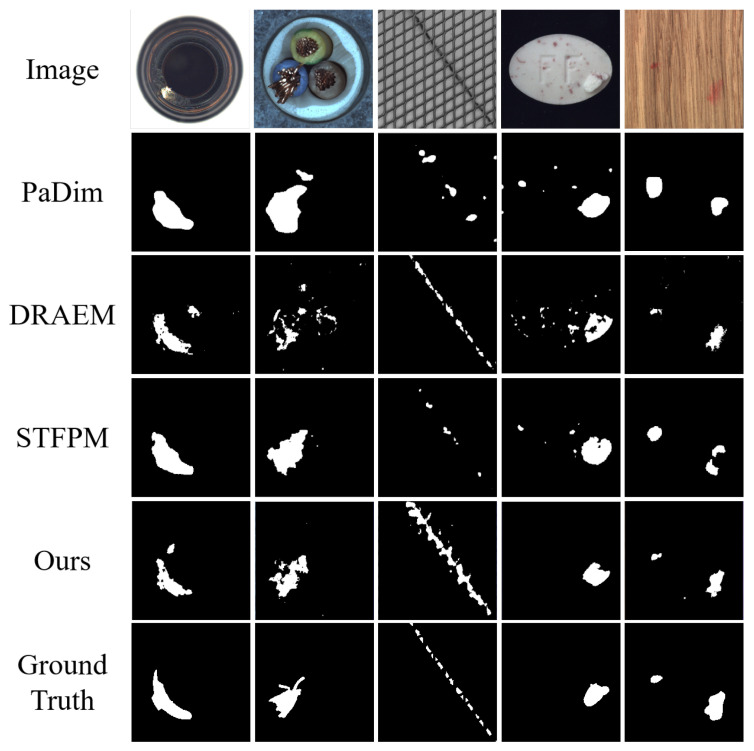
Several examples of comparisons between predicted results from different methods and ground truth images.

Samet Akcay et al. [34] proposed the anomalib library based on the PyTorch Lightning architecture, which includes several state-of-the-art anomaly detection algorithms. We reproduced these anomaly detection algorithms on a computer equipped with an NVIDIA RTX 3090 GPU. The parameter settings for all methods remained consistent with the original papers, and a quantitative comparison was conducted against our proposed algorithm (as shown in the Table 1 and Table 2). Our method achieved the highest AUROC in 14 out of the 15 categories in the dataset, with an average value of 99.70% when rounded to two decimal places. This is 1.1 percentage points higher than the previous best-performing method, and it outperformed the baseline method DRAEM in all aspects. Furthermore, based on the ROC curve, the optimal threshold for distinguishing between defective and non-defective items was determined. The accuracy of defect detection reached 98.41%, with an average inference time of 0.041 s per sample during testing. Moreover, the results demonstrate the exceptional stability of our method on texture-based datasets, with nearly all the values of AUROC approaching 100%, as well as on several datasets of regular-shaped objects. The test results of some categories are shown in Figure 5, and the distribution of predicted defect locations almost coincides with the actual situation. Taking the cable dataset as an example, we show their ROC curves in Figure 6, and it can be seen that the area under the curves is close to 1. Figure 7 are visualizations of box plots for Table 1 and Table 2, which intuitively demonstrate the different distributions of results for various testing methods. Our method has the most concentrated distribution among all methods. Figure 8 displays comparisons between our method and three other methods, PaDim, DRAEM, and STFPM, in terms of predicted and ground truth images for some samples. It can be observed that our method is closer to the ground truth images. The model performs poorly on several types of data, which can be explained by the fact that our defect synthesis method creates abnormal images that are relatively realistic, posing a greater challenge to anomaly detection.

### 4.3. Defect Localization

**Figure 9:**
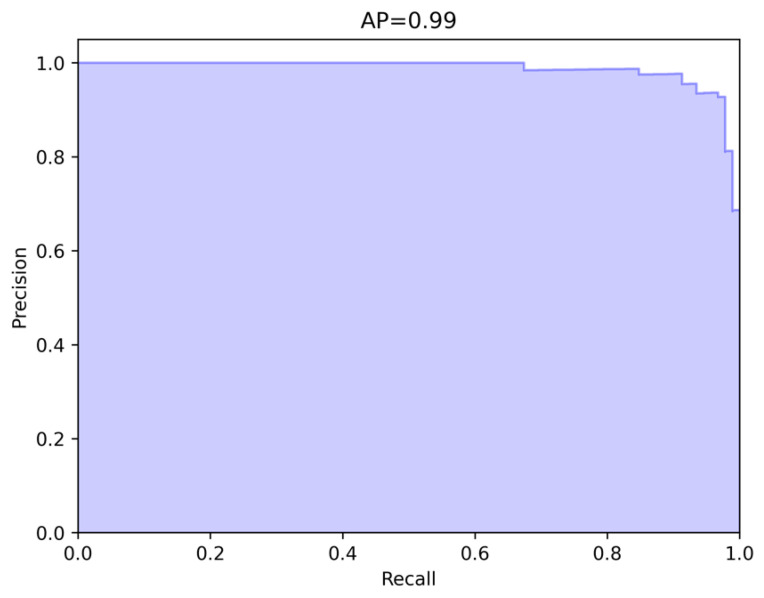
The AP curve for the cable dataset is shown in the upper and lower halves of the figure, respectively.

We compared the performance of our method with several latest pixel-level anomaly detection methods in terms of the AP performance metric (as shown in the Table 3). Our method outperformed the baseline method DRAEM in terms of AP scores in all 15 categories, with a numerical improvement of 31.47%. Our method also surpassed other detection methods (data sourced from DRAEM). We also take the cable dataset as an example and show the obtained AP curve in Figure 9. It can be seen that the precision values can still maintain a relatively high level at high recall rates, indicating that our model can predict the true anomaly distribution accurately after training.

### 4.4. Ablation Experiments

In order to demonstrate the effectiveness of the network structure, we designed several sets of control experiments, mainly evaluating from three aspects: model design, abnormal image source selection, and network training.

#### 4.4.1. Model Structure

We incorporated a deep feature extractor on the basis of the reconstruction network autoencoder and evaluated its impact on anomaly detection. Through comparative experiments (as shown in item 1 and 2 in Table 4), it was found that the reconstruction network, with the addition of the deep feature extractor, had some improvement in detection performance compared to DRAEM. This can be explained by the fact that the addition of deep feature loss makes the reconstructed image and the original input image visually and deeply feature-wise closer, making the information contained in the reconstructed image more abundant and specific.

Next, we fixed the existing autoencoder reconstruction network and conducted comparative experiments on the encoding part of the defect prediction network using the RepLKNet structure, which showed significant improvement in performance compared to the baseline model. This is because the actual receptive field of the larger convolution kernel is larger than the effective receptive field of the stacked small convolution kernels, as proven in the RepLKNet paper. A larger receptive field allows the network to better understand the global structure and contextual information in the image, avoiding overfitting during network training and thus learning more general features in the image.

#### 4.4.2. Abnormal Appearance

**Figure 10:**
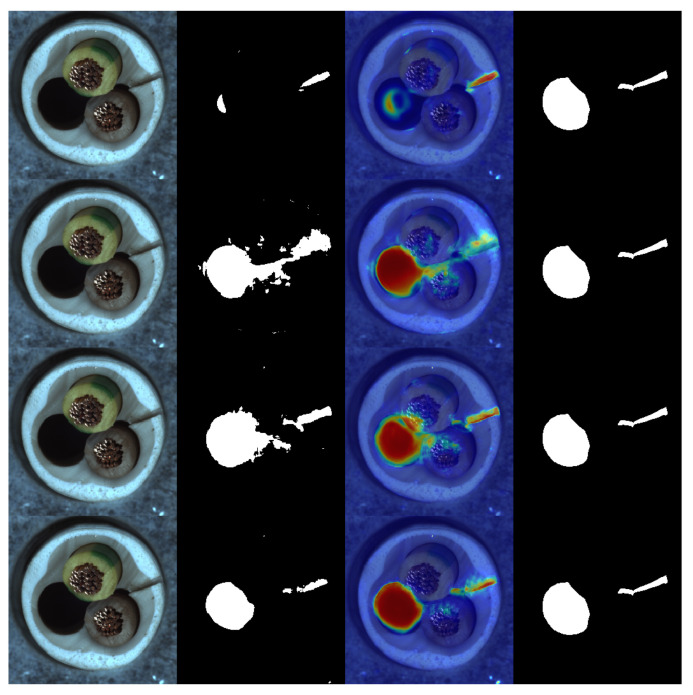
The first row to the last row in the figure are baseline method (item 4 in Table 4), ablation experiment of generative network (item 6 in Table 4), ablation experiment of defect prediction network (item 5 in Table 4), and our method (item 7 in Table 4), respectively. The rightmost column in the image is the ground truth.

We evaluated the proposed new anomaly synthesis method by changing the anomaly source from the DTD dataset used by DRAEM to the MVTec anomaly detection dataset. From the data (as shown in items 1 and 4 in Table 4), it can be seen that this approach slightly improved the detection performance. This may be due to the use of random linear interpolation during the anomaly synthesis process, which allowed the synthesized defective images to retain some of the original image information, allowing the reconstruction network to more accurately recover the original image from these residual information. Furthermore, for some of the object datasets, the defect positions we created accurately appeared on the foreground objects, which is in line with the consensus and allows the network to learn towards discriminating real defects. Under the premise of using the MVTec anomaly detection dataset as the anomaly source, experiments were conducted by adding a deep feature extractor and a large kernel convolution encoder (as shown in items 4, 5, 6, and 7 in Table 4), and the results showed that the network that included all parts (as shown in item 7 in Table 4) had the best performance, confirming the effectiveness and indispensability of the design and composition of the reconstruction network and the defect prediction network. Figure 10 presents examples of performance in each ablation experiment, and it can be observed that our final model displays the results that are closest to the ground truth images.

#### 4.4.3. Training Method

The structure of the deep feature extractor we designed is exactly the same as the encoder part of the reconstruction network, but the training strategy for this part is different from direct training and parameter sharing, instead using a direct copying approach. The experimental results showed that the effect of direct training without parameter copying is comparable to that of the network that only changed the anomaly synthesis method (as shown in items 4 and 8 in Table 4). This suggests that if a similar form of the feature extractor structure is trained directly, it may in turn affect the model’s anomaly detection capability, whereas our parameter copying training method achieved the best results (as shown in items 7 and 8 in Table 4). This is because the autoencoder is constrained by the loss function between the input image and the reconstructed image. After multiple rounds of training, the encoder part learns the ability to extract key feature information from normal or synthesized abnormal input images and uses the decoder to reconstruct the deep features with less data into the original normal image. The feature extraction ability of this encoder is based and unquestionable. Therefore, copying all parameters directly to the deep feature extractor allows it to extract the key features of the reconstructed image, ensuring consistency in deep features between the original and reconstructed images. If the deep feature extractor is directly involved in network parameter updates, the validity of the key information extracted by the extractor will be questioned due to the lack of direct constraints like the reconstruction loss of the autoencoder. Although the deep feature loss correction is used to make the extracted features close to the intermediate layer features of the autoencoder, the cost is that it greatly misleads the network training direction in the early stages of training, making it impossible for the network to converge to the optimal point. This is also one of the factors why the anomaly detection performance of methods such as GANomaly with directly trained feature extractors is not good enough.

## 5. Conclusions

A semi-supervised defect detection algorithm based on defect map prediction with realistic synthetic anomalies is proposed in this paper. Our method demonstrates excellent performance in industrial product defect detection tasks. After conducting experiments on the MVTec dataset, which consists of 15 different categories, our method outperformed other recent detection methods by 1.1 percentage points on the AUROC evaluation metric, showcasing its strong generalization capability. Furthermore, our method surpassed the best-performing DRAEM by 31.5% on the defect localization evaluation metric AP, indicating a significant improvement in localization accuracy. This is because we only learn the distance function between normal and abnormal samples, rather than directly learning the features of anomalies. By employing various data preprocessing techniques such as affine transformations and image enhancement, combined with the utilization of synthetically generated realistic abnormal images as input samples for training, the network has acquired enhanced resistance to interference and robustness. We discussed the design of the two sub-modules, analyzed the benefits of parameter copying in the feature extractor, and demonstrated the effectiveness of large kernel convolution in expanding the receptive field in practical applications via experiments. 

## Figures and Tables

**Table 1:** Our method compared to defect detection algorithms based on optical flow and pre-trained model-based methods: a comparison of AUROC values on the MVTecAD dataset.

Category	Cflow [19]	Csflow [20]	Fastflow [21]	STFPM [22]	RDFOCE [23]	Ours
bottle	**100.0**	99.4	**100.0**	99.8	93.2	99.5
cable	93.1	97.3	90.8	93.4	92.9	**98.8**
capsule	90.3	97.7	87.6	67.5	90.5	**99.5**
carpet	94.8	97.9	97.2	98.4	98.3	**99.8**
grid	86.5	99.3	98.3	93.8	94.7	**100.0**
hazelnut	99.3	93.2	81.0	99.1	**100.0**	**100.0**
leather	99.9	99.7	**100.0**	**100.0**	86.5	**100.0**
metal nut	97.9	94.6	95.7	98.5	97.4	**100.0**
pill	90.2	93.3	91.4	76.7	95.7	**98.8**
screw	91.0	98.1	72.4	79.5	88.6	**100.0**
tile	91.0	98.1	72.4	79.5	88.6	**100.0**
toothbrush	95.0	94.3	82.2	86.3	97.0	**100.0**
transistor	91.4	98.0	91.0	91.8	93.1	**99.2**
wood	99.6	98.7	96.8	98.7	99.2	**100.0**
zipper	92.1	98.6	94.0	84.6	92.7	**99.9**
Average	94.7	97.3	91.6	90.9	93.3	**99.7**

**Table 2:** Our method compared to defect detection algorithms based on feature extraction and image reconstruction methods: a comparison of AUROC values on MVTecAD dataset.

Category	PC * [4]	PaDim [25]	DFM [26]	DRAEM [10]	CFA [27]	Ganomaly [9]	Ours
bottle	**100.0**	99.4	**100.0**	99.2	99.8	54.6	99.5
cable	98.7	84.3	95.6	91.8	97.2	56.6	**98.8**
capsule	97.2	90.1	94.4	98.5	90.7	66.6	**99.5**
carpet	98.1	94.5	81.7	97.0	97.3	55.8	**99.8**
grid	97.0	85.7	73.6	99.9	95.0	86.0	**100.0**
hazelnut	**100.0**	75.0	99.4	**100.0**	**100.0**	88.5	**100.0**
leather	**100.0**	98.2	99.3	**100.0**	**100.0**	43.8	**100.0**
metal nut	99.6	96.1	92.2	98.7	99.1	48.7	**100.0**
pill	94.2	86.3	96.1	98.9	94.9	66.7	**98.8**
screw	97.3	75.9	89.0	93.9	70.8	44.3	**100.0**
tile	98.7	95.0	96.6	99.6	99.8	59.3	**100.0**
toothbrush	**100.0**	88.9	96.9	**100.0**	**100.0**	41.9	**100.0**
transistor	**100.0**	92.0	93.9	93.1	96.5	58.2	**99.2**
wood	99.4	97.6	97.7	99.1	99.5	86.9	**100.0**
zipper	99.4	77.9	96.9	**100.0**	96.7	56.2	**99.9**
Average	98.6	89.1	93.6	98.0	95.8	60.9	**99.7**

* PC refers to PatchCore.

**Table 3:** Our method compared to advanced anomaly localization algorithms: a comparison of AP values on the MVTecAD dataset.

Category	US [35]	RIAD [36]	PaDim	DRAEM	Ours
bottle	74.2	76.4	77.3	86.5	**99.8**
cable	48.2	24.4	45.4	52.4	**99.6**
capsule	25.9	38.2	46.7	49.4	**99.9**
carpet	52.2	52.2	60.7	53.5	**100.0**
grid	10.1	36.4	35.7	65.7	**100.0**
hazelnut	57.8	33.8	61.1	92.9	**100.0**
leather	40.9	49.1	53.5	75.3	**100.0**
metal nut	83.5	64.3	77.4	96.3	**100.0**
pill	62.0	51.6	61.2	48.5	**99.8**
screw	7.8	43.9	21.7	58.2	**100.0**
tile	65.3	52.6	52.4	92.3	**100.0**
toothbrush	37.7	50.6	54.7	44.7	**100.0**
transistor	27.1	39.2	72.0	50.7	**98.9**
wood	53.3	38.2	46.3	77.7	**100.0**
zipper	36.1	63.4	58.2	81.5	**100.0**
Average	45.5	48.2	55.0	68.4	**99.9**

**Table 4:** Ablation experiments on different comparison schemes.

	Structure		Abnormal Appearance		Training Approach		Result	
Number	Deep Features	Large Kernel	MVTec AD	DTD	Parameter Copying	Gradient Update	AUROC	AP
1				✓			98.00	68.40
2	✓			✓			99.23	99.62
3		✓		✓			99.61	99.78
4			✓				99.25	99.54
5		✓	✓				99.58	99.79
6	✓		✓		✓		99.27	99.49
7	✓	✓	✓		✓		99.70	99.87
8	✓	✓	✓			✓	99.33	99.69

## Data Availability

This study analyzed the MVTec anomaly detection public dataset, which can be found at https://www.mvtec.com/company/research/datasets/mvtec-ad (accessed on 17 July 2023).
